# Performance evaluation of sperm concentration, motility, and morphological analysis for GSA‐810 series of sperm quality analysis system

**DOI:** 10.1002/jcla.24986

**Published:** 2023-11-27

**Authors:** Yan‐Mei Ge, Jin‐Chun Lu, Shan‐Shan Tang, Yuan‐Hua Xu, Yuan‐Jiao Liang

**Affiliations:** ^1^ Center for Reproductive Medicine, Zhongda Hospital Southeast University Nanjing Jiangsu China

**Keywords:** computer‐assisted sperm analysis system, performance evaluation, sperm concentration, sperm morphology, sperm motility

## Abstract

**Background:**

The performance evaluation of each computer‐assisted sperm analysis (CASA) system may provide a basis for the interpretation of clinical results and further improvement of the CASA system.

**Methods:**

The accuracy of the GSA‐810 CASA system was evaluated by detecting latex bead quality control products. The precision of sperm concentration, morphology, and percentages of progressively motile sperm (PR) were evaluated by coefficient of variation (CV). Three samples with sperm concentration of about 100 × 10^6^/mL were diluted to evaluate the linear range.

**Results:**

The detection values of latex beads were within the range of target values. The CVs of sperm concentration and PR were significantly and negatively correlated with sperm concentration (*r* = −0.561, *p* = 0.001) and PR value (*r* = −0.621, *p* < 0.001), respectively. The *R* ^2^ values of the linear range of sperm concentration were ≥0.99. There was no significant difference in sperm motility and PR within 1–10 min at 36.5°C ± 0.5°C. The coincidence rates of sperm morphology and sperm head morphology for 36 semen samples analyzed by the GSA‐810 system and manual method were 99.40% and 99.67%, respectively. The CVs of the percentage of sperm with abnormal morphology and percentage of sperm with abnormal head morphology were less than 5%.

**Conclusion:**

The GSA‐810 system can accurately analyze normal semen samples, but the repeatability of the results is poor for oligozoospermia and asthenozoospermia samples. The future CASA system for analyzing sperm morphology should focus on recognizing the middle and tail segments of a spermatozoon.

## Graphical abstract

The GSA‐810 system can accurately detect sperm concentration in semen samples with sperm concentrations in the range of (2–100) × 10^6^/mL. Semen samples with a sperm concentration between 50 × 10^6^/mL and 100 × 10^6^/mL can be directly detected using the GSA‐810 system without dilution. The higher the sperm concentration and PR, the better the repeatability of the detection results.

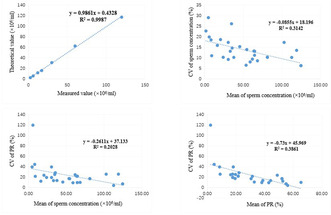

## 1 INTRODUCTION

Semen analysis is one of the most basic tests used to assess male fertility and provide reference for diagnosis of infertility and observation of efficacy. Manual microscope observation was the main method for semen analysis. However, its detection items were limited, and its operation was complicated. Especially when evaluating sperm motility, there was greater subjectivity. Moreover, it had poor repeatability.^1^, ^2^ In recent years, with the development of reproductive medicine, a computer‐assisted sperm analysis (CASA) system has become the main means of semen analysis in reproductive medicine laboratories because of its high accuracy, quantitative data of sperm dynamic parameters, and rapid detection. Currently, the utilization rate of CASA in Chinese laboratories has reached 92%.^3^

However, most CASA systems allow partial automation in routine sperm analysis.^4^ Moreover, the existing CASA systems still have some defects.^5^ Their analysis accuracy is poor at lower and higher sperm concentrations. Different CASA systems use different algorithms to calculate sperm quality parameters, such as sperm concentration, sperm motility, percentage of progressively motile sperm (PR), sperm dynamic parameters, and sperm morphology parameters, which may lead to differences in semen analysis results. In addition, the lack of quality control and standardized procedures has also affected the clinical application of CASA.^4^, ^5^

With the continuous development of science and technology, CASA systems based on artificial intelligence (AI) are expected to improve the efficiency of analysis and the accuracy of detection results. The AI‐based CASA system integrates artificial intelligence algorithms and autofocusing optical system to analyze sperm quality parameters. Compared with the existing CASA system, it is more portable and easy to use, and the detection results are highly correlated with those obtained by manual semen analysis.^6^ Previous studies have shown some differences in sperm motility between the manual analysis and a CASA system.^6^ In a recent study, the AI‐equipped LensHooke® X1 PRO system was shown to be able to detect sperm concentrations over a very wide range (0.1–300 × 10^6^/mL). Moreover, it showed greater sensitivity and specificity (>90%) in identifying samples of oligospermia and asthenospermia compared with the IVOS II system.^7^ The continuous improvement in the performance of AI‐based CASA systems may be the development direction of semen analysis automation in the future.

Nevertheless, before entering clinical application, the performance of each CASA system should be evaluated to provide a basis for the interpretation of clinical results. The performance evaluation will also provide a reference for further improvement of the CASA system. Although some studies^8^, ^9^, ^10^, ^11^ have compared and analyzed the detection results of semen samples from different CASA systems, these studies either lack quality control measures or only evaluate a single parameter, making it difficult to evaluate the performance of a CASA system with the results obtained.

The GSA‐810 series of sperm quality analysis system (GSA‐810 system) uses neural network based image recognition technology to identify sperm, uses the optical flow method to track sperm targets, and then obtains a series of measurement parameters by classification, positioning, and detection of sperm targets, thereby grading sperm motility and classifying sperm morphology. This study evaluates the performance of the GSA‐810 system in analyzing sperm concentration, motility, and morphology, with a view to providing a reasonable explanation of the detection results when it enters clinical application, offering a basis for improving its performance, and supplying a reference for the performance evaluation of other CASA systems used in the clinics.

## 2 MATERIALS AND METHODS

### 2.1 Source of specimens

A total of 86 outpatients treated in our center for infertility between June 2022 and November 2022 were randomly selected. After abstinence for 2–7 days, semen samples were collected from each patient by masturbation and put into an aseptic semen collection cup. After routine examination, the remaining semen samples were used in this study. Samples of azoospermia and extremely severe oligozoospermia (<2 × 10^6^/mL) were excluded for high sampling error associated with low sperm numbers.^12^ This study was conducted in the Center for Reproductive Medicine of Zhongda Hospital Affiliated to Southeast University, which was approved by the Reproductive Medicine Ethics Committee of Zhongda Hospital affiliated to Southeast University (Reproduction No. 2015‐1), and all patients signed the informed consent.

### 2.2 Instruments, technicians, and reagents

The GSA‐810 system and sperm concentration quality control products (latex bead suspensions) were provided by Hua Yue Medical Technology Co., Ltd. (Guangzhou, China). The system was made of a NIKON® Eclipse Ci microscope with phase contrast, equipped with a IP103100A digital camera, a motorized stage system, a heating platform and a GSA‐810 image acquisition software (Microptic SL). The camera had 3.15 megapixels and shot at 85 frames per second. The nominal values for high and low concentration of latex bead suspensions were (80.00 ± 8.0) × 10^6^/mL and (15.00 ± 1.5) × 10^6^/mL, respectively. Laboratory technicians had over 10 years of work experience and had received full training before using the CASA system. Diff‐Quik staining solution was purchased from Zhuhai Beisuo Biological Technology Co., Ltd. (Zhuhai, Guangdong, China).

### 2.3 Quality control for sperm concentration

It has been reported that latex beads are commonly used as quality control materials to evaluate the accuracy and stability of sperm concentration.^13^, ^14^ In this study, high and low concentration of latex bead suspensions were first analyzed 10 times repeatedly with the GSA‐810 system to test whether the results were in control and calculate their coefficients of variation (CV). After that, the latex bead quality control materials were tested before each detection to ensure the results were in control.

### 2.4 Performance evaluation of sperm concentration and motility detected by the GSA‐810 system

#### 2.4.1 Linear range of sperm concentration detected by the GSA‐810 system

Three semen samples with sperm concentration of about 100 × 10^6^/mL were selected and diluted with their own seminal plasma for 2, 4, 7, 10, 20, and 50 times, respectively. Each concentration point of each sample was detected three times, and the mean value was calculated. A correlation curve was drawn based on the measured value and theoretical value, and the *R* ^2^ value was calculated. The theoretical value refers to the value that should be obtained based on the original sperm concentration and dilution ratio.

#### 2.4.2 Repeatability analysis of sperm concentration and motility

Thirty fresh semen samples were collected. Sperm concentration and motility were analyzed 10 times repeatedly for each sample using the GSA‐810 system. The mean value and CV for each sample were calculated. The average CV values of different sperm concentration groups (sperm concentration < 15 × 10^6^/mL, [15–50] × 10^6^/mL, and >15 × 10^6^/mL) and different PR groups (PR ≤32% and PR >32%) were compared. The correlations of the mean value of sperm concentration with the CV values of sperm concentration and PR were analyzed.

#### 2.4.3 Effects of temperature on sperm motility at different time points

Twenty fresh semen samples were collected randomly. The temperature control platform was kept at 36.5°C ± 0.5°C. Sperm motility of each sample was detected by the GSA‐810 system once every minute within 1–10 min to observe the influence of temperature on sperm motility and determine the number of samples that can be loaded continuously.

### 2.5 Performance evaluation of sperm morphology analyzed by the GSA‐810 system

#### 2.5.1 Accuracy evaluation of sperm morphology analyzed by the GSA‐810 system

Sperm smears from 36 semen samples were stained using Diff‐Quik staining solution. The staining method had been validated to be effective compared to the Papanicolaou staining method in human sperm.^15^ Sperm morphology was analyzed by the GSA‐810 system and manual method, respectively. When using the GSA‐810 system to analyze sperm morphology, a sperm smear was loaded onto the platform and dropped with immersion oil. Then, the GSA‐810 system automatically focused on the sperm, captured sperm images, and analyzed them. At least 200 sperm were captured for each sample, and a maximum of 100 fields of view could be observed. For the manual method, sperm morphology analysis were performed by a technician under oil immersion at 1000 × magnification.

The coincidence rate between them was calculated according to the following formula: (A1 + B1)/(A + B) × 100%. A1: The number of sperm with normal morphology identified by the artificially confirmed; B1: The number of sperm with abnormal morphology identified by the artificially confirmed; A: The number of sperm with normal morphology identified by the GSA‐810 system; B: The number of sperm with abnormal morphology identified by the GSA‐810 system.

#### 2.5.2 Accuracy evaluation of sperm head morphology analyzed by the GSA‐810 system

Normal sperm head morphology is the most important factor in sperm morphology analysis. Therefore, we also compared the coincidence rate of sperm head morphology analyzed by the GSA‐810 system and manual method, respectively, which was calculated according to the following formula: (N1 + T1)/(N + T) × 100%. N1: The number of sperm with normal head morphology identified by the artificially confirmed; T1: The number of sperm with abnormal head morphology identified by the artificially confirmed; N: The number of sperm with normal head morphology identified by the GSA‐810 system; T: The number of sperm with abnormal head morphology identified by the GSA‐810 system.

#### 2.5.3 Repeatability evaluation of sperm morphology analyzed by the GSA‐810 system

Five semen samples were randomly selected, and sperm smears were stained using Diff‐Quik staining solution. Sperm morphology was analyzed 10 times repeatedly for each sample using the GSA‐810 system. The percentage of sperm with abnormal morphology, the percentage of sperm with abnormal head morphology, and their respective CV values were calculated.

### 2.6 Statistical analysis

The sample size required for this study meets the requirements of relevant literature and performance verification.^16^, ^17^, ^18^, ^19^ The data were analyzed by statistical software SPSS 25.0 (SPSS Inc., Chicago, IL, USA). The correlations between sperm concentration, PR, and their CV values were analyzed by Pearson or Spearman correlation analysis tests. Due to the normal distribution of the data, Pearson correlation analysis was conducted. The comparisons of sperm motility and PR at different time points were performed by paired *t*‐test. Chi‐square analysis was used to compare the percentages of sperm with normal morphology and percentages of sperm with abnormal head morphology between the GSA‐810 system and manual method. *p* ≤ 0.05 was considered to be statistically significant.

## 3 RESULTS

### 3.1 The accuracy and repeatability of latex bead quality control products detected by the GSA‐810 system

High and low concentration of latex bead suspensions were detected 10 times repeatedly by the GSA‐810 system, and their values were (79.24 ± 5.93) × 10^6^/mL and (15.12 ± 1.06) × 10^6^/mL, respectively, and were within the range of target values. Their CV values were 7.48% and 7.01%, respectively.

### 3.2 Linear range of sperm concentration detected by the GSA‐810 system

**FIGURE 1:**
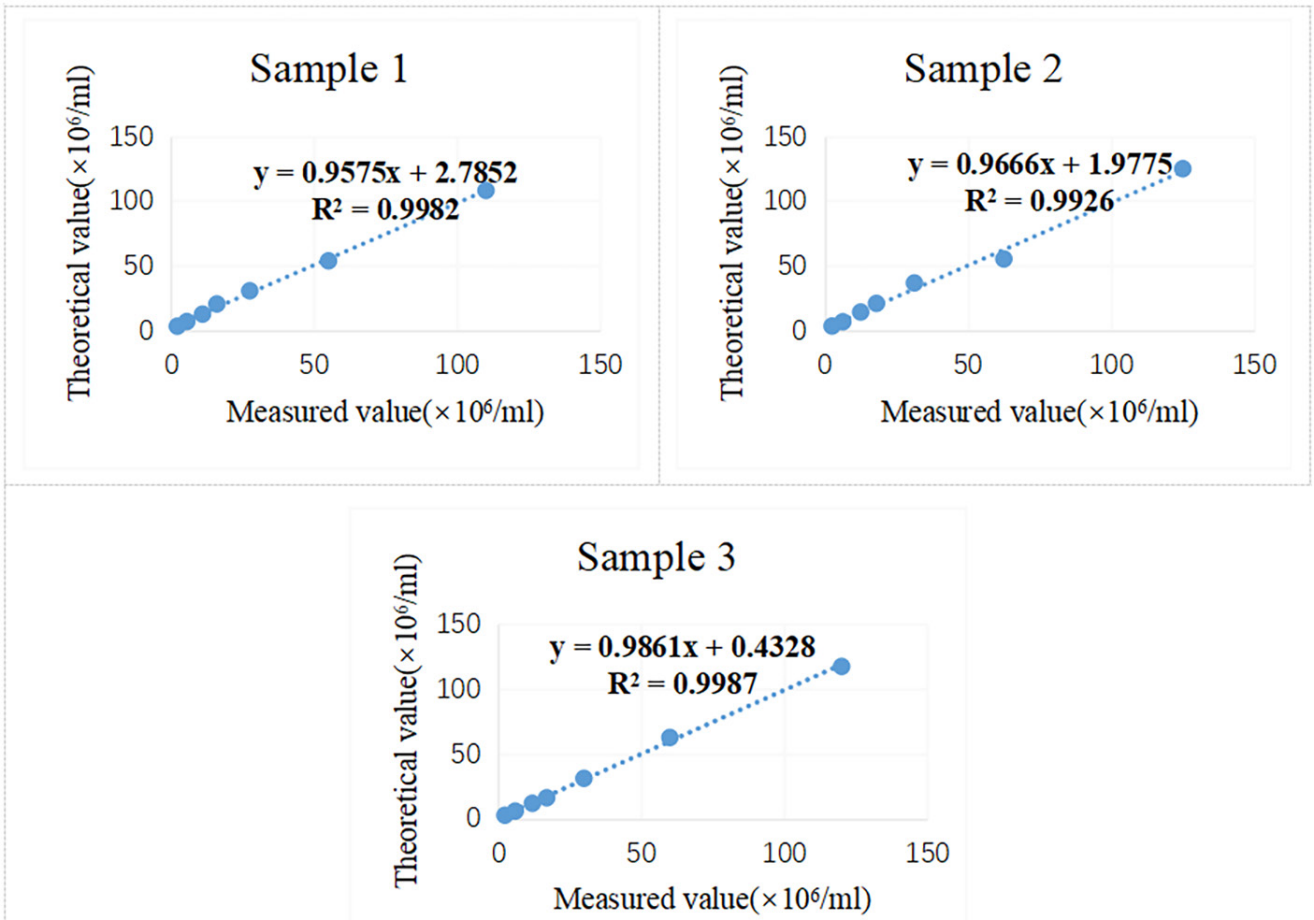
Linear range of sperm concentration detected by the GSA‐810 system. Three semen samples with sperm concentration of about 100 × 10^6^/mL were diluted with their own seminal plasma for 2, 4, 7, 10, 20, and 50 times, respectively. Each concentration point of each sample was detected three times by the GSA‐810 system, and the mean value was calculated. A correlation curve was drawn based on the measured value and theoretical value, and the *R* ^2^ value was calculated. All *R* ^2^ values were ≥0.99.

The correlation curve for each sample based on the measured value and theoretical value was shown in Figure 1, and all *R* ^2^ values were ≥0.99.

### 3.3 Repeatability analysis of sperm concentration and PR

**FIGURE 2:**
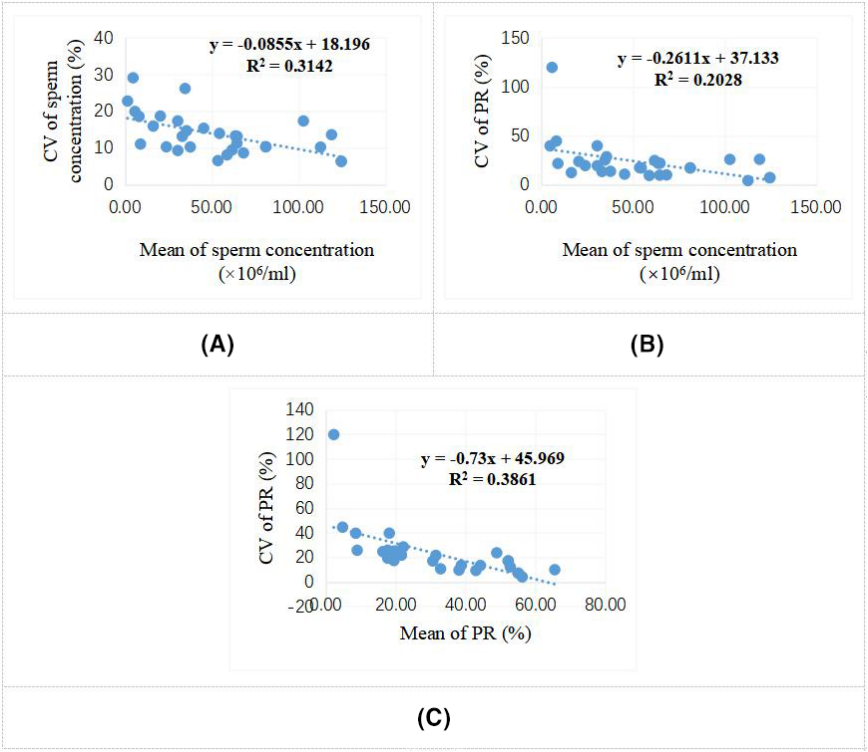
Correlations of the CV values of sperm concentration and PR with the mean values of sperm concentration and PR. CV, coefficients of variation; PR, percentage of progressively motile sperm. Sperm concentration and PR of 30 fresh semen samples were analyzed 10 times repeatedly for each sample using the GSA‐810 system. The mean value and CV for each sample were calculated. The correlations of the mean value of sperm concentration with the CV values of sperm concentration and PR were analyzed. (A) The CV of sperm concentration was significantly and negatively correlated with sperm concentration (*r* = −0.561, *p* = 0.001). (B) The CV of sperm PR was significantly and negatively correlated with sperm concentration (*r* = −0.439, *p* = 0.017). (C) The CV of sperm PR was significantly and negatively correlated with sperm PR value (*r* = −0.621, *p* < 0.001).

As sperm concentration increased, the CV value of sperm concentration gradually decreased. The average CV values at sperm concentrations <15 × 10^6^/mL (*n* = 5), (15–50) × 10^6^/mL (*n* = 10), and >50 × 10^6^/mL (*n* = 15) were 20.28%, 15.11%, and 10.58%, respectively. As shown in Figure 2, the CV of sperm concentration was significantly and negatively correlated with sperm concentration (*r* = −0.561, *p* = 0.001).

Repeatability analysis of sperm PR showed that the average CV values at PR ≤32% (*n* = 16) and PR >32% (*n* = 14) were 31.23% and 11.90%, respectively. As shown in Figure 2, the CV of sperm PR was significantly and negatively correlated with sperm concentration (*r* = −0.439, *p* = 0.017) and sperm PR value (*r* = −0.621, *p* < 0.001), especially with sperm PR value.

### 3.4 Effects of temperature on sperm motility at different time points

Our results (Table 1) showed that within 10 min of semen samples being placed on the temperature control platform of the GSA‐810 system, there were no significant changes in sperm motility and PR. The continuous loading of eight semen samples by the GSA‐810 system will not affect the analysis results of sperm motility and PR.

**TABLE 1:** Comparisons of sperm motility and PR at different time points.

Variable	Detection time point									
	1 min	2 min	3 min	4 min	5 min	6 min	7 min	8 min	9 min	10 min
Sperm motility	45.66 ± 18.38	44.06 ± 18.08	44.00 ± 17.00	43.25 ± 17.03	43.42 ± 16.97	44.78 ± 16.68	42.87 ± 16.21	43.07 ± 16.63	41.98 ± 16.44	41.91 ± 16.99
*p*		0.783	0.768	0.669	0.691	0.874	0.614	0.642	0.508	0.506
PR	38.22 ± 15.70	35.50 ± 13.82	35.33 ± 13.14	35.83 ± 13.91	34.49 ± 13.23	36.61 ± 13.98	35.15 ± 12.99	35.29 ± 13.78	34.36 ± 13.59	34.41 ± 13.22
*p*		0.564	0.532	0.613	0.422	0.733	0.505	0.534	0.411	0.412

*Note*: PR: percentage of progressively motile sperm. Sperm motility and PR of each of 20 fresh semen samples were detected by the GSA‐810 system once every minute within 1–10 min. The data were expressed as mean ± standard deviation. Paired *t*‐test was used to compare the results at each time point and 1 min, and *p* values were given.

### 3.5 Performance evaluation of sperm morphology analysis

The coincidence rates of sperm morphology and sperm head morphology for 36 semen samples analyzed by the GSA‐810 system and manual method were 99.40% and 99.67%, respectively. There were no significant differences in the percentage of sperm with normal morphology ([1.78 ± 2.31]% vs. [1.38 ± 1.48]%, *p* = 0.561) and percentage of sperm with abnormal head morphology ([97.20 ± 2.96]% vs. [97.18 ± 2.59]%, *p* = 1.000) between the GSA‐810 system and manual method. The repeatability analysis of sperm morphology showed that the CVs of the percentage of sperm with abnormal morphology and percentage of sperm with abnormal head morphology were less than 5%, and that their average CVs were less than 3% (Table 2).

**TABLE 2:** Repeatability analysis of sperm morphology.

Sample	Number of analyses	Percentage of sperm with abnormal morphology (%)	CV (%)	Percentage of sperm with abnormal head morphology (%)	CV (%)
1	10	96.1 ± 3.7	3.89	95.1 ± 4.3	4.47
2	10	85.4 ± 3.4	4.02	82.5 ± 3.1	3.77
3	10	98.1 ± 0.9	0.93	97.8 ± 1.0	1.04
4	10	99.8 ± 0.3	0.26	99.5 ± 0.5	0.53
5	10	91.6 ± 1.8	1.99	89.2 ± 1.8	2.02
Average value of CV (%)			2.22		2.37

*Note*: Five semen samples were randomly selected, and sperm smears were stained using Diff‐Quik staining solution. Sperm morphology was analyzed 10 times repeatedly for each sample using the GSA‐810 system. The percentage of sperm with abnormal morphology, the percentage of sperm with abnormal head morphology, and their respective CV values were presented. The results showed that their CVs were less than 5%, and that their average CVs were less than 3%.

## 4 DISCUSSION

Sperm quality analysis systems or CASA systems have been widely used in reproductive medicine and andrology laboratories in China. Compared with the manual method, a CASA system is more objective, accurate, rapid, and has better repeatability in analyzing semen samples, especially in the analysis of sperm motility. However, the detection results of different CASA systems may vary greatly due to their different detection principles, frame rates, parameter settings, and configurations.^20^, ^21^, ^22^ Therefore, it is particularly important to evaluate the performance of a CASA system prior to clinical application. So, how do you evaluate the performance of a CASA system? What parameters need to be evaluated? In this study, we attempted to evaluate the performance of the GSA‐810 system with AI features in analyzing sperm concentration, motility, and morphology.

First, we evaluated the accuracy of the GSA‐810 system in detecting sperm concentration using quality control materials such as latex beads. The results showed that the values of high and low concentration of latex bead suspensions detected by the GSA‐810 system were within the range of target values, and that their CV values were less than 10%. It is suggested that the accuracy of the GSA‐810 system in detecting sperm concentration could meet clinical requirements. Next, we evaluated the linear range of sperm concentration analyzed by the GSA‐810 system, and the results showed that the correlation coefficients of the three samples in the range of (2–100) × 10^6^/mL were greater than 0.99, suggesting that the GSA‐810 system could accurately detect sperm concentration in semen samples with sperm concentrations in the range of (2–100) × 10^6^/mL. The WHO manual recommends that sperm concentrations of between 2 × 10^6^/mL and 50 × 10^6^/mL can be measured by a CASA, and that samples with a sperm concentration higher than 50 × 10^6^/mL will need to be diluted.^12^ Our results show that semen samples with a sperm concentration between 50 × 10^6^/mL and 100 × 10^6^/mL can be directly detected using the GSA‐810 system without dilution. However, it is still necessary to further evaluate whether semen samples with a sperm concentration higher than 100 × 10^6^/mL need to be diluted.

The repeatability results of sperm concentration and PR showed that with the increase of sperm concentration and PR values, their CV values gradually decreased. When sperm concentration was below 15 × 10^6^/mL, the average CV value reached 20.28%. When sperm PR was below 32%, the average CV value reached 31.23%. It is suggested that when the sperm concentration and PR of semen samples were low, the repeatability of detection results was poor. Jin reported that the CV value of sperm concentration detected by a CASA system was less than 5%.^23^ But only one semen sample was detected 20 times repeatedly, and its sperm concentration was as high as 170.11 × 10^6^/mL. Another study reported that the repeatability of sperm concentration and motility detected by the BEION S3 sperm quality analyzer in four semen samples with different sperm concentrations was good, and that all of CV values were less than 15%.^8^ However, in their study, three of the four samples had sperm concentrations higher than 50 × 10^6^/mL, and the lowest sperm concentration also reached 15.3 × 10^6^/mL, with a CV value of 14.38%. It was also reported that there was increased variability in sperm concentration results of semen samples with low‐ (<15 × 10^6^/mL) and high sperm concentrations (>60 × 10^6^/mL) detected by a CASA system, and that the assessment of sperm motility were inaccurate in samples with higher sperm concentration, non‐sperm cells and fragments.^24^ The high variability of a CASA system, including the GSA‐810 system used in this study, in detecting semen samples with low sperm concentration and motility may be attributed to the following reasons. First, the detected semen sample is not well mixed, which may result in a small portion of the sample being taken during each sampling not representing the overall sample. Second, there are fragments, gel‐like particles, or non‐sperm cells in some semen samples, which may interfere with sperm count and motility analysis. Third, the number of spermatozoa counted is limited. When the total number of spermatozoa counted is 400, the sampling error is 5%.^12^ With the decrease of counted spermatozoa, the sampling error gradually increases. Fourth, the depth of a counting chamber on different brands of counting chambers may vary. If the depth of a counting chamber is inaccurate or different depths of counting chambers such as 10 or 20 μm deep are used, the results of sperm concentration and PR may be different.^25^, ^26^ Fifth, the factors of a CASA system itself, such as whether it is equipped with an ordinary microscope or a phase contrast microscope, whether there is a stable constant temperature stage, the quality of hardware and software for sperm recognition and analysis, and the setting of parameters, can all lead to differences in the analysis results of different CASA systems.^27^ Last, the factors of the operators, such as whether they are careful, whether the operation is standardized, and whether the sample size loaded each time is consistent, can all lead to variations in the detection results. With the integration of different artificial intelligence technologies, development of tracing technology, and obvious increase in the number of sperm analyzed each time in the future, it is expected to reduce the variability of detection results.

Continuous analysis after loading multiple semen samples at once can improve clinical detection efficiency, which is very beneficial for clinical medical institutions with a large number of patients. The GSA‐810 system can continuously load eight different semen samples or four samples loaded twice each. Currently, there are few reports on the impact of the temperature control platform of a CASA system on sperm motility. In our study, we found that there were no significant changes in sperm motility and PR detection results of the semen samples on the temperature control platform within 10 min, which may be related to the relatively constant temperature in the box structure of the GSA‐810 system. It was indicated that the GSA‐810 system could continuously load 8 semen samples, which may greatly improve the detection efficiency of clinical semen samples.

Due to the heterogeneity of sperm morphology within a single sample or multiple samples from the same subject, sperm morphology is the most difficult parameter to evaluate and the least reliable parameter.^28^ Currently, there are few studies on the performance evaluation of a CASA system for sperm morphology. Schubert et al.^2^ evaluated the performance of a CASA system in detecting sperm morphology, but they used David's modified criteria^29^ instead of the strict criteria recommended by the World Health Organization (WHO).^12^ One study^30^ indicated that the correlation between the two morphological classification systems was poor and that they lacked comparability. In this study, we evaluated the sperm morphology of 36 semen samples using the GSA‐810 system and the manual method, respectively, based on the criteria recommended by the WHO. The results showed that there were no significant differences in the percentage of sperm with normal morphology and percentage of sperm with abnormal head morphology between the GSA‐810 system and manual method. Moreover, the CVs of the percentage of sperm with abnormal morphology and percentage of sperm with abnormal head morphology were less than 5%. The high coincidence rate and good repeatability between the GSA‐810 system and manual method were mainly related to the vast majority of abnormal sperm in the analysis of sperm morphology, while the GSA‐810 system was more accurate in identifying abnormal sperm. However, the GSA‐810 system was difficult to recognize sperm between normal and abnormal conditions and could not accurately identify the neck, middle, and principal piece of a spermatozoon. Therefore, when using the GSA‐810 system for the analysis of sperm morphology, manual correction is necessary for sperm that has been recognized to be normal. The future CASA system for analyzing sperm morphology should focus on identifying the middle and tail segments of a spermatozoon.

## 5 CONCLUSIONS

There are significant differences in the detection performance of different CASA systems. In order to ensure the comparability of the results detected by CASA systems from the same laboratory or different laboratories, it is necessary to perform a performance evaluation of each sperm analysis system before it enters clinical use, and fully understand the accuracy and clinical significance of relevant performance parameters. The GSA‐810 system could accurately analyze normal semen samples, but the repeatability of the results was poor for oligozoospermia and asthenozoospermia samples. The GSA‐810 system could accurately identify abnormal sperm heads, but it had poor accuracy in identifying sperm between normal and abnormal conditions, as well as the neck, middle, and principal piece of a spermatozoon. Due to the lack of a standard method for evaluating the performance of a CASA system, our performance evaluation of the GSA‐810 system may not be comprehensive. We hope that in the future, relevant organizations will develop performance evaluation methods and standards for a CASA system to ensure the comparability of the results detected by different CASA systems, thereby providing reliable evidence‐based medical evidence for patients' disease diagnosis, treatment plan selection, and therapeutic efficacy monitoring.

## AUTHOR CONTRIBUTIONS

Jin‐Chun Lu and Yuan‐Jiao Liang conceived the idea and directed the writing of our manuscript. Yan‐Mei Ge performed the experiment, analyzed the data and wrote the manuscript. Shan‐Shan Tang and Yuan‐Hua Xu performed the experiment and analyzed the data. All authors had made a substantial, direct and intellectual contribution to the work, and approved it for publication.

## FUNDING INFORMATION

This work received no support or any funding from any source.

## CONFLICT OF INTEREST STATEMENT

The authors declare that they have no competing interests.

## CONSENT TO PARTICIPATE

All patients signed the informed consent.

## Data Availability

The data sets used and/or analyzed during the current study are available from the corresponding author on reasonable request.
